# Methallylsulfonate Polymeric Antiscalants for Application in Thermal Desalination Processes

**DOI:** 10.3390/polym16192838

**Published:** 2024-10-08

**Authors:** Ali A. Al-Hamzah, Christopher M. Fellows, Osman A. Hamed

**Affiliations:** 1 Water Technologies Innovation Institute & Research Advancement, Saudi Water Authority, Al Jubail 31961, Saudi Arabia; aal-hamzah2@swcc.gov.sa (A.A.A.-H.); oahmed@yahoo.com (O.A.H.); 2 School of Science and Technology, The University of New England, Armidale, NSW 2351, Australia

**Keywords:** thermal desalination, calcium sulfate, scale inhibitor, seawater desalination

## Abstract

Nine copolymers of acrylic acid and sodium methallyl sulfonate were tested as scale inhibitors in thermal desalination. The nine antiscalants covered molar masses between 2000 and 9500 g.mol^–1^ and concentrations of sulfonated monomer ranging between 10 and 30 mole percent. A pressure measurement and control (P-MAC) unit and a high-temperature pressurized vessel were used to measure the effectiveness of the scale inhibitors in seawater, concentrated seawater, and model solutions at 125 °C. The effectiveness of the novel copolymers was comparable to commercial antiscalant at times up to 15 min and improved at longer times. Molar mass was a more important determinant of effectiveness than degree of sulfonation, with the greatest mitigation of calcium sulfate precipitation observed for antiscalants of molar mass 2000 to 2500 g.mol^–1^ regardless of sulfonate content. Antiscalants of molar mass 4500 to 5000 g.mol^–1^ showed a higher threshold effect than antiscalants of molar mass 7000 to 9500 g.mol^–1^, with a 30% sulfonated polymer of molar mass 4500 g.mol^–1^ performing appreciably better than other polymers of a similar molar mass.

## 1. Introduction

While the relative contribution of thermal desalination to global water production from seawater has declined significantly over the past few decades, in absolute terms it has only recently plateaued, and Multi-Stage Flash (MSF) and Multiple-Effect Distillation (MED) still provide the majority of the potable water for the Gulf Co-operation Council region, where the total dissolved solids content of seawater is significantly higher than that of standard seawater [1]. The factors that have led to the replacement of thermal desalination by membrane-based processes are purely economic [2], with thermal processes being significantly less vulnerable to negative variations in water quality, to defects in operation and maintenance, and to supply chain disruptions. The formation of inorganic scale, chiefly ‘hard scale’ composed of calcium sulfate, has a negative impact on the operation of thermal desalination plants by reducing heat exchanger effectiveness [1,3]. As calcium sulfate is an inverse solubility salt, its precipitation is the primary factor determining the top brine temperature of operations [4,5,6,7,8,9]. As the overall efficiency of thermal desalination increases with increasing top brine temperature, more effectively addressing the problem of inorganic scaling in thermal desalination is important. This is true not only for improving the efficiency of the operation of existing thermal desalination infrastructure, but for improving the economic competitiveness of potential future thermal desalination plants operating over an extended temperature range. The primary means by which hard scale formation is controlled is by the addition of scale inhibitors at the low-ppm level, typically polycarboxylate polymers of relatively low molar mass [10,11,12,13,14,15,16]. Carboxylates have been demonstrated to interact with forming calcium sulfate microcrystallites not only to inhibit precipitation but to enhance the formation of calcium sulfate hemihydrate at low temperatures and are not necessarily the ideal polymeric species for calcium sulfate control [17]. We have previously reported on the synthesis by Reversible Deactivation Radical Polymerization and testing of poly(acrylic acid) scale inhibitors incorporating various end groups on the formation of calcium sulfate scale under conditions relevant to thermal desalination [18]. The work reported on here, which was carried out previous to that work but has only become possible to publish at this time, deals with the testing of copolymers of acrylic acid and sodium methallyl sulfonate as scale inhibitors. In contrast to carboxylates and phosphonates, relatively little published work has appeared on the use of the sulfonate functional group in scale inhibition (e.g., [19,20,21]) and none to our knowledge on the prevention of hard scale under the conditions prevailing in thermal desalination. Due to the similarity in structure between the sulfonate group and the sulfate group, it is reasonable to expect relatively strong interactions between sulfonate and anion binding sites on the surface of forming calcium sulfate crystallites, impeding growth of such crystallites, and for this reasons sulfonate antiscalants have found application in the control of barium sulfate scaling in oilfields [19,20].

## 2. Materials and Methods

### 2.1. Copolymere

Nine copolymers of acrylic acid and sodium methallyl sulfonate (SMS) were synthesized in Degussa Laboratories in Krefeld, Germany, and supplied as viscous solutions of 29.9–31.5% solids. The major known characteristics of the copolymers as reported by Degussa Laboratories are given in Table 1.

^1^H (Figure S2) and ^13^C (Figure S1) NMR of samples 2, 4–7, and 9 were collected on a Spinsolve 80 spectrometer (Magritek GmbH, Aachen, Germany) in aqueous solution and are shown in the Supplementary Information. Spectra showed a qualitative increase in peaks associated with SMS consistent with the reported composition, although the broad overlapping polymer peaks could not be integrated to give a quantitative composition.

Size Exclusion Chromatography was carried out on samples 1–2, 4–7, and 9 with a 1260 Infinity system (Agilent Technologies, Dubai, United Arab Emirates) using two PL Aquagel-OH 20 columns in series with both UV and RI detection (Figure S3). Samples of approximately 2 mg/mL were prepared in 0.34 M NaCl solution buffered with 1% *w/v* 1:1 H_2_PO_4_^−^:HPO_4_^2−^. A calibration curve was prepared using five molar mass standards of poly(acrylic acid) (Agilent; M_p_ = 1550, 2925, 7500, 16,000, and 28,000 g/mol). The eluagrams obtained are shown in the Supplementary Information and poly(acrylic acid) equivalent molar masses are given in Table 1 for both RI and UV eluagrams.

### 2.2. Other Materials

Arabian Gulf Seawater of approximate composition given below (Table 2) and artificial seawater were employed. AR-grade sodium chloride, potassium chloride, magnesium sulfate, calcium chloride, and sodium bicarbonate were used to prepare artificial seawater.

The supersaturation index (SI) for calcium sulfate hemihydrate was calculated for solutions of relatively low ionic strength using the expression derived by Shen et al. [22], and for ionic strengths greater than or equal to seawater the SI was calculated using the Pitzer model [23] for ion activities and the calcium sulfate hemihydrate solubility data of Zannoni et al. [24].

Total dissolved solids (TDS) was determined by gravimetry (ASTM D 1193), pH by potentiometry using a standard hydrogen electrode (ASTM D1293, AWWA Standard Method 4500-H+ B), chloride by titration with silver nitrate (ASTM D 512, AWWA Standard Method 4500 B), bromide by ion chromatography (ASTM D 6581, AWWA Standard Method #4110 B), calcium, magnesium, potassium, and sodium by inductively coupled plasma optical emission spectroscopy (AWWA Standard Method 3120B), and hydrogen carbonate by titration with hydrochloric acid (ASTM D 3875-15).

### 2.3. Description of Experimental Units

The bench-top tests were carried out in two separate units. Preliminary tests were first carried out in the pressure measurement and control (P-MAC) unit (P-MAC Systems, Aberdeen, United Kingdom). A high-temperature–high-pressure test unit constructed in the WTIIRA engineering workshop was then utilized for the screening tests.

#### 2.3.1. Pressure Measurement and Control (P-MAC) Test Unit

**Figure 1:**
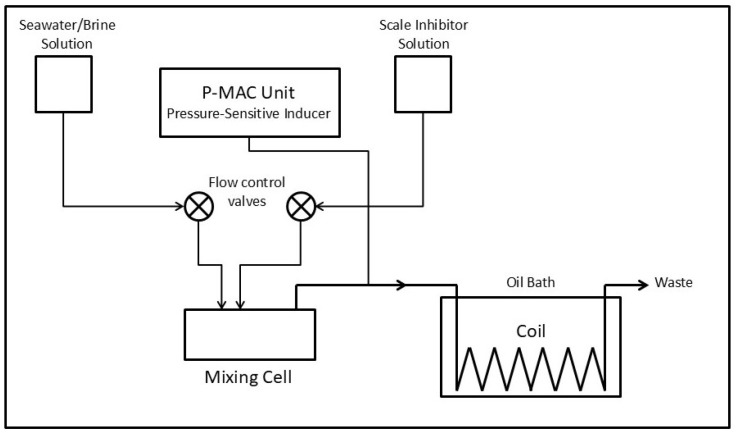
Schematic of pressure measurement and control (P-MAC) unit.

Brine and scale inhibitor solution were separately pumped at constant flow rates of 10 mL/min with HPLC piston pumps into a mixing cell and the solution was then passed through a 1.016 mm ID stainless steel 316 capillary tube immersed in an oil bath heated to 125 °C (Figure 1). Any scale deposition reduces the bore of the tube and causes an increase in pressure drop across the cell. The rate of change in pressure across the coil was monitored via a ceramic pressure sensor inside the P-MAC unit. The resultant signal was conditioned and amplified to produce a buffered output suitable for displaying as a scaling curve on an external chart recorder. Each test was carried out until a differential pressure of 2.0 psi was obtained. This relatively low pressure, correlated with a low degree of scaling on the coil surface, was chosen to aid in cleaning of the coil. Cleaning of the coil after calcium sulfate deposition was carried out by sequential passage of 5% disodium ethylenediamine tetraacetic acid and 5% HCl solutions at room temperature.

Note that due to the requirement to obtain observable scaling effects for observation and comparison, this work was carried out at temperatures and concentrations above those used in commercial MSF plants, where the concentration of brine is rarely above 1.4 times that of seawater and temperatures are held well below 122 °C (where SI ~1.0 for a concentration factor of 1.4).

#### 2.3.2. High Temperature-High Pressure Test Unit

**Figure 2:**
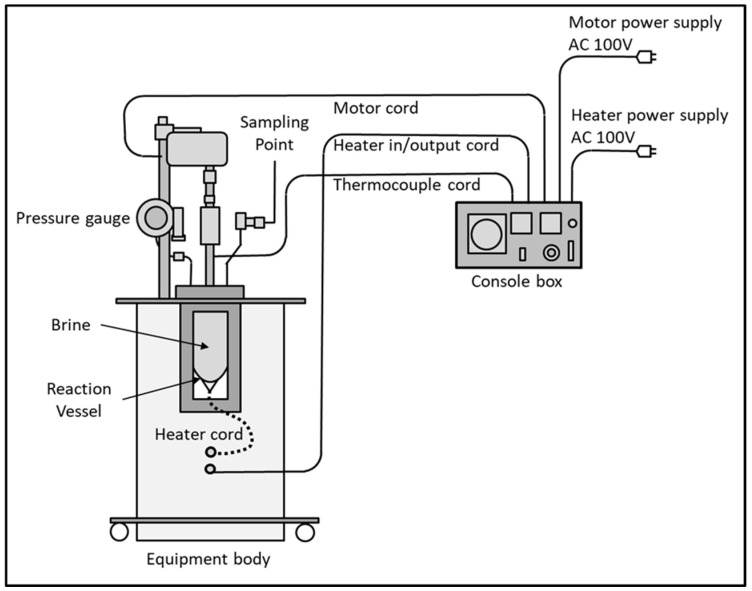
Schematic of high-temperature high-pressuretest unit.

A high temperature-high pressure test unit was constructed in order to evaluate the threshold effect of the antiscalants (Figure 2). The unit consisted of a glass reaction vessel with a heating coil molded inside its walls. The vessel was covered at the top by a stainlesssteel lid equipped with a stirrer, a thermocouple to sense the temperature, a pressure gauge, and a sampling point. The rate of heating and stirring was controlled by a separate control unit. The test unit is capable of handling chemical reactions at conditions up to 350 °C and 0.3 barg. An amount of 470 mL of test solution was poured into the reaction flask. The lid was tightly clamped to the flask in order to avoid any leakage. The solution was heated slowly with stirring, which was fixed at 300 rpm. Within around 20 min, the solution temperature reached 125 °C. The moment at which the temperature reached 125 °C was considered time zero. The rate of deposition was followed by monitoring the loss in calcium concentration with time. A series of 50 mL samples were collected at 5 min intervals for a period of 30 min for this purpose and filtered with a 0.45 μm filter. Calcium determination on the filtered samples was performed using a Metrohm Microprocessor (model 796).

## 3. Results

Three consecutive series of tests were carried out. In the first series, preliminary evaluation tests were carried out to identify the composition of the brine solution that was compatible with the response and performance of the pressure measurement and control (P-MAC) unit. In the second series, as a result of the technical problems which were experienced with the P-MAC unit, intensive evaluation and screening tests were performed on the high-temperature–high-pressure test unit available in the WTIIRA laboratories. In the third series, screening tests were carried out using the P-MAC unit based on the test results of the second series.

### 3.1. Series One: Preliminary Evaluation Tests on the P-MAC Unit

Preliminary evaluation tests were first performed on the P-MAC unit, with the primary objective of this series being to determine the concentration of the brine solution most appropriate for use in the P-MAC unit. In the first step, brine solutions prepared from seawater with twice (800 ppm Ca^2+^, 6500 ppm SO_4_^2−^, SI = 2.34) and three times (1200 ppm Ca^2+^, 9750 ppm SO_4_^2−^, SI = 6.16) the concentration of typical Arabian Gulf seawater were tested. It was observed that the formation of scale in the capillary tube was delayed for a period of more than 60 min in both cases. In the second step, synthetic brine solutions of different compositions and concentration ratios were tested. The time requirement for the P-MAC unit to reach the targeted pressure drop of 2.0 psi ranged between 90 and 16 min. However, at low scaling times which were obtained through the use of more concentrated solutions, calcium sulfate precipitates were observed forming in the preparation vessel.

To eliminate the possibility of salt precipitation during the preparation of highly concentrated brine solutions before they were pumped to the P-MAC unit, the cationic and anionic components of the selected brine solution were separated in two separate vessels into calcium chloride and sodium sulfate. The two solutions were then simultaneously pumped and mixed in the pipe system of the P-MAC well ahead of the heated capillary tube. Synthetic seawater of a concentration factor of two (SI = 6.16) was tested and scale precipitation occurred after around 40 min.

To induce more rapid precipitation, a solution consisting of calcium, sulfate, and bicarbonate with double the concentration of normal sea water and with a small quantity of sodium and chloride ions was tested (SI = 2.86). Precipitation of the scale occurred rapidly in two to three minutes. The more rapid scale formation at the lower SI was attributed to the role of co-precipitating CaCO_3_ in the nucleation of crystal growth. It was concluded that this procedure was the most appropriate for use in the P-MAC unit due to the formation of scale in a reasonable time.

### 3.2. Series Two: Evaluation Tests on the High-Temperature–High-Pressure Unit

Four groups of experiments were carried out. In the first group of experiments, a direct comparison between the threshold effects of the nine antiscalants was carried out using a solution composed from calcium as calcium chloride and sulfate as sodium sulfate dissolved in distilled water. In the second group of experiments, the impact of additional sodium, chloride, bicarbonate, and magnesium ions on the threshold effects of the antiscalants was investigated. In the third group of this series, the best-performing antiscalants which were screened out in the first group of experiments were further tested using a synthetic brine solution composed of all constituents responsible for scale formation, and in the fourth group these antiscalants were tested using concentrated sea water.

#### 3.2.1. Series 2, Group 1: Direct Comparison of the Performances of the Nine Antiscalants

**Figure 3:**
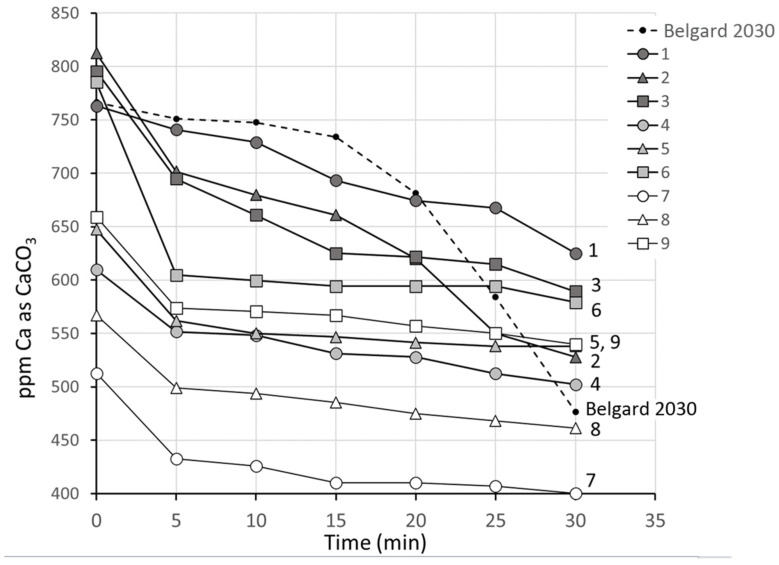
Reduction in calcium concentration in filtrate in the presence of copolymer antiscalants of average molar mass M_m_ 2000–2500 (1–3), 4500–5000 (4–6) and 7000–9500 (7–9) and commercial antiscalant Belgard 2030.

A synthetic brine solution with only calcium and sulfate ions at close to double their concentrations in normal Arabian Gulf seawater (800 ppm Ca^2+^, 6500 ppm SO_4_^2−^) gave good conditions for discrimination (SI = 2.98). Sodium, chloride, and magnesium ions were found to delay the precipitation of calcium sulfate significantly, as expected from theory, and for this reason they were excluded from test solutions [23]. A concentration of 10 ppm of the antiscalant solutions (4.0 to 4.5 ppm on solids basis) was used to provide discrimination between the performance of the different antiscalants. The results of the experiments are shown in Figure 3.

It can be seen in Figure 3 that the initial filtrable calcium concentrations vary greatly. Many of the antiscalants have a negative effect at room temperature, reducing the filterable calcium concentration relative to the reference. This effect scales with molar mass, being most significant for the highest molar mass samples (7–9) and absent for the lowest molar mass samples (1–3). Within a molar mass range, this effect is greatest for the polymers with the lowest sulfonated content (4, 7) and least for the polymers with the highest sulfonated content (6, 9). The cause of this reduction in filterable calcium is unknown but it is possibly due to the formation of insoluble calcium polyacrylate under transient conditions of high antiscalant concentration which can then nucleate the growth of calcium sulfate. This incompatibility of polyacrylic acids with calcium ions is well established at concentrations as low as 5 ppm for the polymer and is known to be more significant for homopolymers than copolymers [25]. However, such a phenomenon would only be expected to be significant in solutions of relatively low ionic strength [26].

In the first five minutes of treatment, all antiscalants can be seen to be equally effective (or ineffective), with drops in filtrable calcium of 22–181 ppm compared to 15 ppm for the control experiment. The calcium tolerance of polyacrylic acid has been reported to be inversely proportional to temperature over a relatively small temperature range (25–45 °C) [25,27], so it is possible that the differences between the incompatibilities of the antiscalants have been flattened over this time interval and they are all contributing to nucleation.

At times greater than five minutes, all antiscalants appear to show a positive effect. All but the lowest molar mass copolymers (1–3) have similar performance to the commercial antiscalant within the first 15 min, and all show better performance than the commercial product as tests continue beyond 15 min. The decline in filtrable calcium between 5 and 30 min was 275 ppm for the commercial antiscalant Belgard 2030, 105–175 ppm for the antiscalants of M_m_ 2000–2500, 25–50 ppm for the antiscalants of M_m_ 4500–5000, and 30–40 ppm for the antiscalants of M_m_ 7000–9500. Within a molar mass range, there was no clear trend in behavior with degree of sulfonation. This finding suggests that all species used were effective in retarding further growth of calcium sulfate nuclei by adsorbing to crystal growth sites [28], but that the lowest molar mass cohort of scale inhibitors was more susceptible to desorption under the conditions of the experiments. Considering all measured calcium loss, however, the set of lower molar mass antiscalants showed the best inhibition behavior.

#### 3.2.2. Series 2, Group 2: Impact of Other Ions on Antiscalant Performance

**Figure 4:**
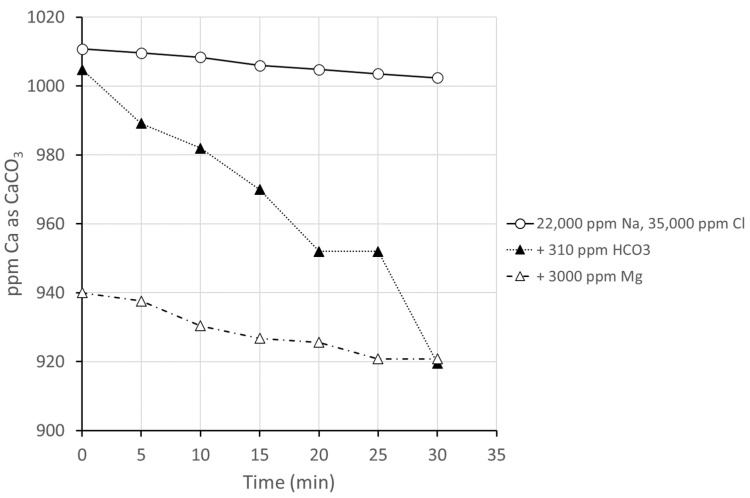
Reduction in calcium concentration in filtrate in the presence of low-molar-mass scale inhibitor 1 (9% SMS, M_m_ = 2500).

In the second group of experiments, the role of other ions on the performance of a representative antiscalant was investigated under conditions where initial calcium was held constant at 1000 ppm and initial sulfate at 6500 ppm. Three conditions were investigated: (a) total concentration of the matrix with sodium chloride corresponding to approximately twice that of normal Arabian Gulf seawater, with 22,000 ppm sodium and 46,000 ppm chloride (SI = 2.0); (b) addition to (a) of sodium bicarbonate, giving a bicarbonate concentration of 310 ppm, approximately double that of standard Arabian Gulf seawater; and (SI = 2.0) (c) addition to (b) of magnesium as magnesium chloride to give a magnesium ion concentration of 3000 ppm, approximately double that found in standard Arabian Gulf seawater (Figure 4) (SI = 2.2). The pH of the tested solutions (b) and (c) was measured between 9.2 and 9.3.

It can be seen that in comparison with the results for systems only containing the scale-forming ions (Figure 3), the performance of the inhibitor is much improved under the higher ionic strength conditions; this is not surprising, considering the strong dependence of the calcium sulfate solubility product on ionic strength [20]. The reduced performance in the presence of bicarbonate is consistent with the apparent accelerating effect of calcium carbonate in scale nucleation seen in the preliminary P-MAC investigations (Section 3.2.1). The initial drop in calcium concentration in the presence of a high amount of magnesium is unexpected; it is possible that the increase in overall hardness with the addition of such a large amount of magnesium (equivalent to over 12,000 ppm CaCO_3_) leads to precipitation of insoluble polyacrylate salts. Disregarding the change in the calcium concentration at time = 0, it is clear that the performance of the scale inhibitor over the rest of the test is only slightly impaired by the addition of magnesium.

This synthetic brine containing calcium, sulfate, bicarbonate, magnesium, sodium, and chloride ions at concentrations double those of seawater was used as a medium for further discrimination of the low-molar-mass antiscalants 1–3 and 6.

#### 3.2.3. Series 2, Group 3: Comparison of Antiscalants on Synthetic Concentrated Seawater

**Figure 5:**
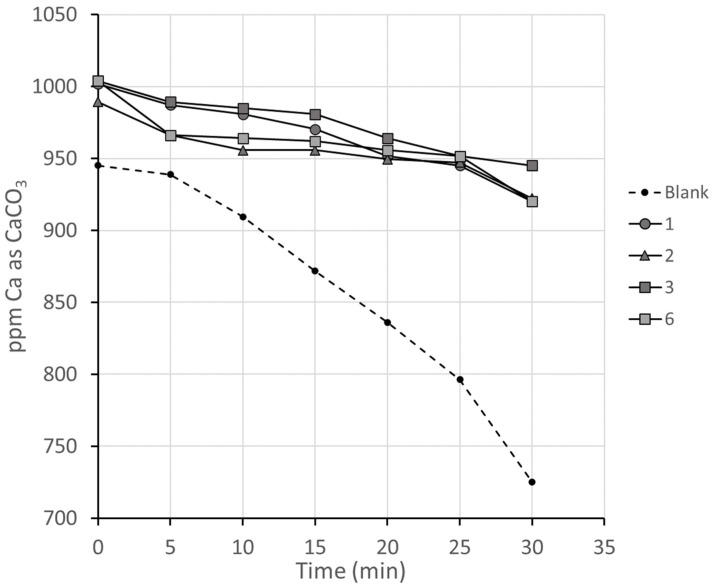
Reduction in calcium concentration in filtrate of concentrated artificial seawater in the presence of antiscalants 1–3 (M_m_ 2000–2500) and 6 (M_m_ 4500).

In this group of experiments, the four antiscalants 1–3 and 6 were tested at a 10 ppm concentration in the synthetic concentrated seawater prepared and a blank test was included for reference (Figure 5).

All four antiscalants were effective in delaying the precipitation of CaSO_4_ compared to the blank solution and the drop before heating previously seen in the presence of magnesium (Figure 4) was not repeated, suggesting that result may have been artifactual. All four antiscalants showed a comparable threshold effect, with differences falling almost within experimental error. This group of experiments did not provide a clear guide for the discrimination between the four competing antiscalants. For 10 min, the approximate maximum residence time of brine in a large MSF system, the reduction in Ca concentration in the presence of the antiscalants was between 15 and 50% of that seen in the absence of any antiscalant.

#### 3.2.4. Series 2, Group 4: Comparison of Antiscalants in Concentrated Seawater

**Figure 6:**
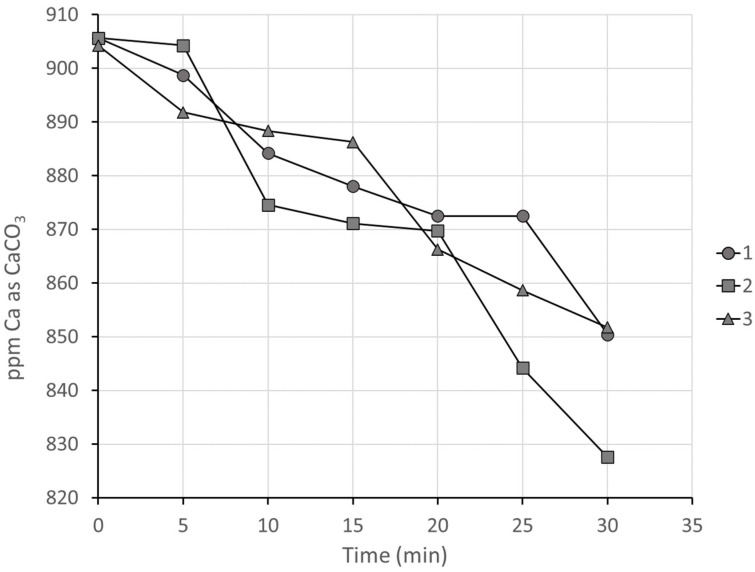
Reduction in calcium concentration in filtrate of concentrated seawater in the presence of antiscalants 1–3 (M_m_ 2000–2500).

As a last trial in the high-temperature–high-pressure test unit, real Arabian Gulf seawater concentrated in the laboratory to approximately double the initial concentration was used for further screening between the three lowest molar mass antiscalants (1–3). The pH of approximately 9 L of seawater was adjusted to 4.5 with diluted hydrochloric acid to destroy all the bicarbonate. The seawater was evaporated at 95 °C to half its original volume. Just before each experiment, the alkalinity of the concentrated brine was readjusted to double its concentration in seawater by the addition of sodium bicarbonate. The threshold performances of the three antiscalants were to a large extent comparable to each other (Figure 6) and there was no clear trend with degree of sulfonate content, paralleling the results obtained in artificial seawater (Figure 5). Overall, the results were extremely similar to the results obtained in artificial seawater, with an average reduction in Ca concentration over 30 min of 6.8% in seawater compared to 6.9% in artificial seawater.

The lack of any strong trend in inhibition behavior with sulfonate content at high-ionic-strength conditions suggests that the lower amount of sulfonate groups are sufficient to target the antiscalants to growth sites on crystallites and that the additional sulfonate content is superfluous.

### 3.3. Series Three: Performance of Low Molar Mass Antiscalants in the P-MAC Unit

Antiscalants 1–3 and 6 were then evaluated further in the P-MAC unit under aggressive operating conditions. A synthetic brine solution with concentrations of calcium, sulfate, and bicarbonate twice those of normal seawater was used as a feed to the P-MAC unit operating at a 125 °C temperature, as per Figure 1. Equal volumes of calcium and sulfate solutions were supplied to the instrument. All cations except sodium were fed as the chloride salts, and all anions except chloride (including the antiscalant) were fed as the sodium salts.

In an initial round of experiments, the optimum dose rate of antiscalant which would induce precipitation in the P-MAC unit in a reasonable time was determined. The effectiveness of antiscalant 1 was tested using dose rates of 10, 5.0, 2.0, and 1.0 ppm. At dose rates of 10 and 5.0 ppm, no scale formation was observed, while at a dose rate of 2.0 ppm, scale precipitation was observed after 26 min. Further reduction in antiscalant dose rate to 1.0 ppm resulted in rapid scale formation and was similar in behavior as the blank solution. A dose rate of 2.0 ppm was therefore selected for use in the P-MAC unit.

For each antiscalant, a wide variation was found in the time to reach the targeted pressure drop of 2.0 psi (Table 3). This variation is concerning and suggests that the method used could lead to the retention of nuclei for scale formation on the surface of the test coil.

While there is clearly a great degree of uncertainty in the P-MAC results, they are correlated positively with the drops in [Ca^2+^] between 5 minutes and 30 min shown in Figure 3, Figure 5, and Figure 6.

## 4. Conclusions

A series of polycarboxylate antiscalants containing 10–30 weight% sulfonated monomer, methallyl sulfonate, were tested for their ability to retard calcium sulfate formation in a simple solution of calcium and sulfate ions at 125 °C and under conditions approximating those found in high-temperature Multi-Stage Flash (MSF) thermal desalination of seawater. At high-ionic-strength conditions, copolymers with M_m_ between 2000 and 4500 were shown to be most effective, inhibiting calcium sulfate formation with up to 85% effectiveness over the time scale required for MSF for realistic scale-forming solutions. No clear trend in effectiveness with sulfonate content was observed at high ionic strength, suggesting that extending the studies to copolymers with a lower fraction of allyl sulfonate comonomer may be valuable. At low-ionic-strength conditions, lower molar mass copolymers and copolymers with higher sulfonate content appeared to be more effective. Further work on the system is required in order to clarify the structure–property relationships and the role of the sulfonate group in the inhibition of calcium sulfate scaling.

## Figures and Tables

**Table 1:** Composition and molar mass of antiscalants.

Sample Number	*w*/*w* % SMS	Molar Mass/10^3^ (M_m_) Degussa/RI/UV	Molar Mass/10^3^ (M_n_)
1	9	2.5/2.9/2.3	1.6/1.5/1.2
2	18	2.0/2.6/1.8	1.5/1.5/0.9
3	28	2.5//	1.6//
4	9	5.0/5.9/3.0	3.0/3.4/1.3
5	19	5.0/5.5/4.3	3.0/2.5/1.7
6	28	4.5/5.0/3.4	3.0/2.1/1.6
7	9	9.0/10.9/8.7	6.0/6.5/2.9
8	19	9.5//	6.0//
9	30	7.0/8.7/5.8	5.0/5.7/2.0

**Table 2:** Representative Arabian Gulf seawater composition [6].

Parameter	Value	Unit
TDS	44,500	ppm
pH	8.1	
Chloride (Cl^−^)	23,500	ppm
Sodium (Na^+^)	12,400	ppm
Sulfate (SO_4_^2−^)	3290	ppm
Magnesium (Mg^2+^)	1530	ppm
Calcium (Ca^2+^)	450	ppm
Potassium (K^+^)	470	ppm
Hydrogen carbonate (HCO_3_^−^)	98	ppm

**Table 3:** P-MAC results for antiscalants 1–3 and 6 for feed solution containing 2.0 ppm antiscalant solution, 1000 ppm Ca^2+^ 6500 ppm SO_4_^2−^, 22,000 ppm Na^+^, 35,000 ppm Cl^−^, 310 ppm HCO_3_^−^, and 3000 ppm Mg^2+^.

Antiscalant	1	2	3	6
Time to reach pressure drop of 2 psi (min)	26.5	28.5	45	8.2
	14.37	28	7.03	3.02
	11.18	13	4.75	3
	5.5	18.5	4.02	3
	4.0	7.83	2.33	2.82
	3.37	7.78	2.03	2.23
	3.0	6.5		2.06
	2.93	3		2
	2.72	2.33		
	2.5			
	2.02			
Average	7.1	11.7	10.9	3.3
Standard Deviation	7.2	9.3	15.4	1.9
Maximum	26.5	28.5	45	8.2

## Data Availability

Data are available from the corresponding author upon request.

## Supplementary Materials

The following supporting information can be downloaded at: https://www.mdpi.com/article/10.3390/polym16192838/s1, Figure S1: 13C NMR of copolymers; Figure S2: 1H NMR of copolymers; Figure S3: Size Exclusion Chromatrography eluagrams; (a) RI detector; (b) UV detector.
