# Supplementary material for: Methallylsulfonate Polymeric Antiscalants for Application in Thermal Desalination Processes
Source: Polymers (Basel). 2024 Oct 8;16(19):2838. doi: 10.3390/polym16192838 (PMC11478755; doi:10.3390/polym16192838)
Supplement: Supplementary file 1 [file polymers-16-02838-s001.zip › polymers-3246752-supplementary.pdf]

Supplementary Information

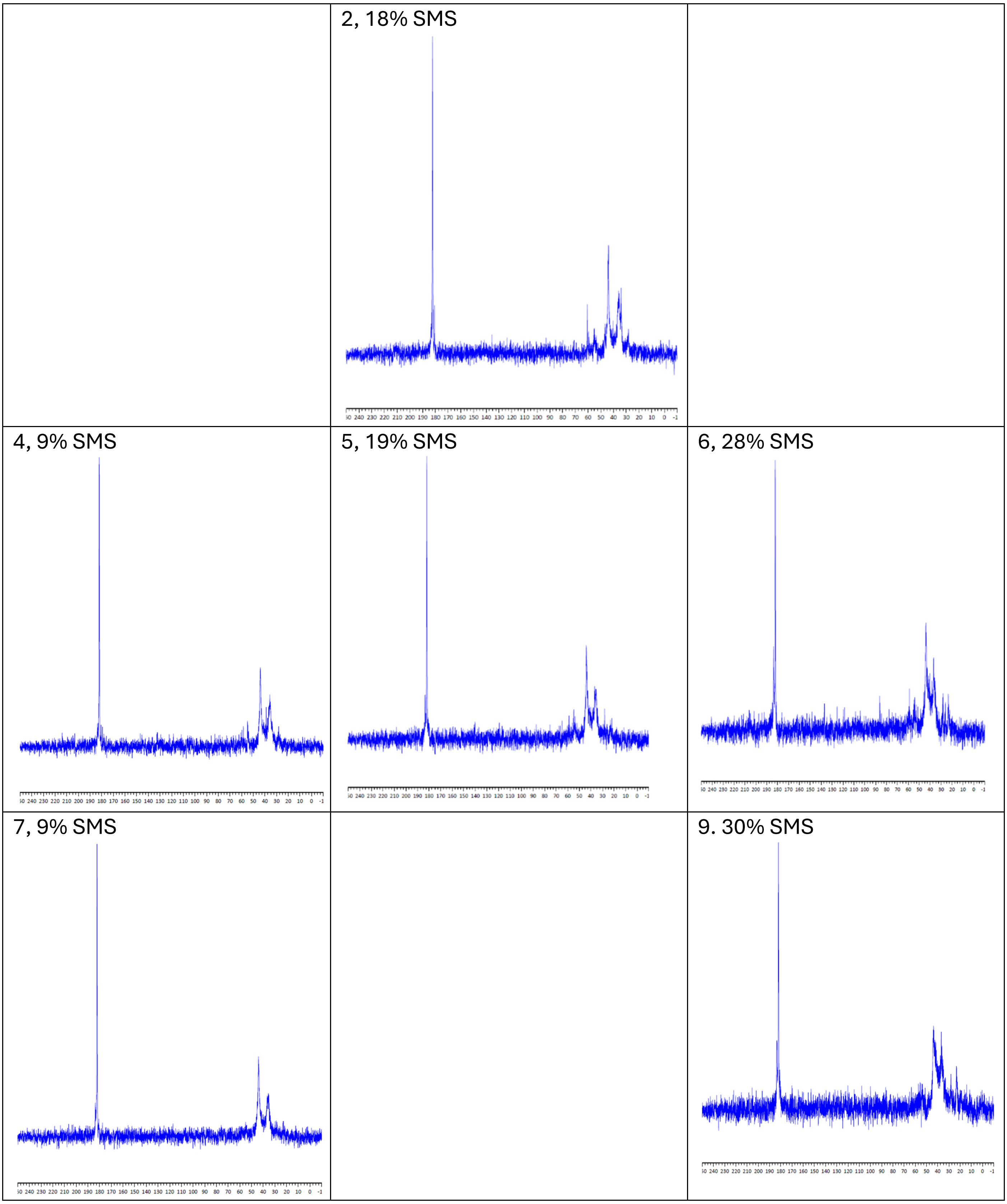

Figure S1: 13C NMR of copolymers

	2, 18% SMS 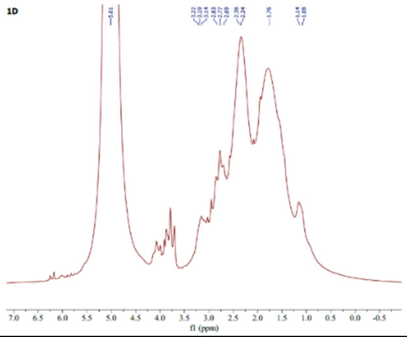	
4, 9% SMS 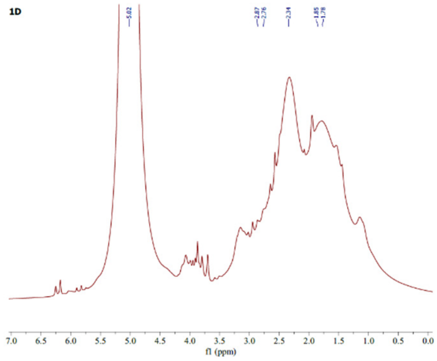	5, 19% SMS 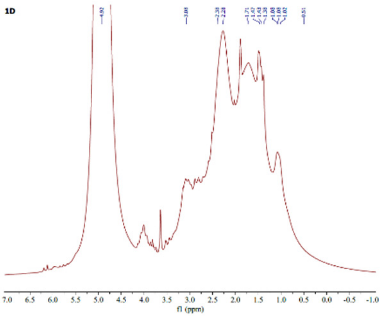	6, 28% SMS 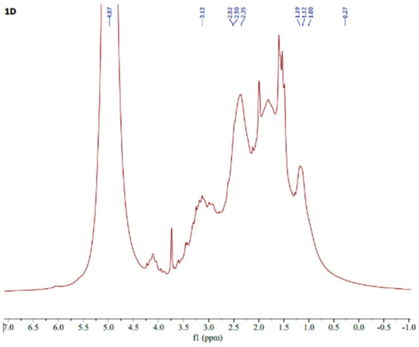
7, 9% SMS 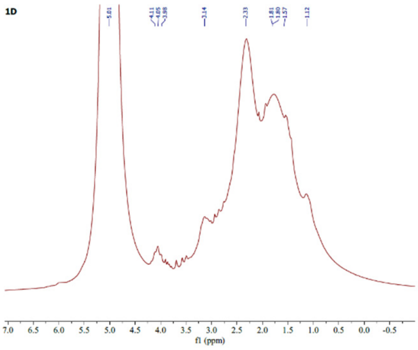		9. 30% SMS 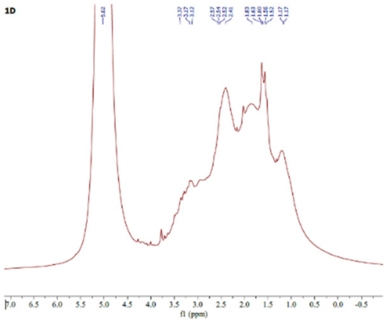

Figure S2: 1H NMR of copolymers

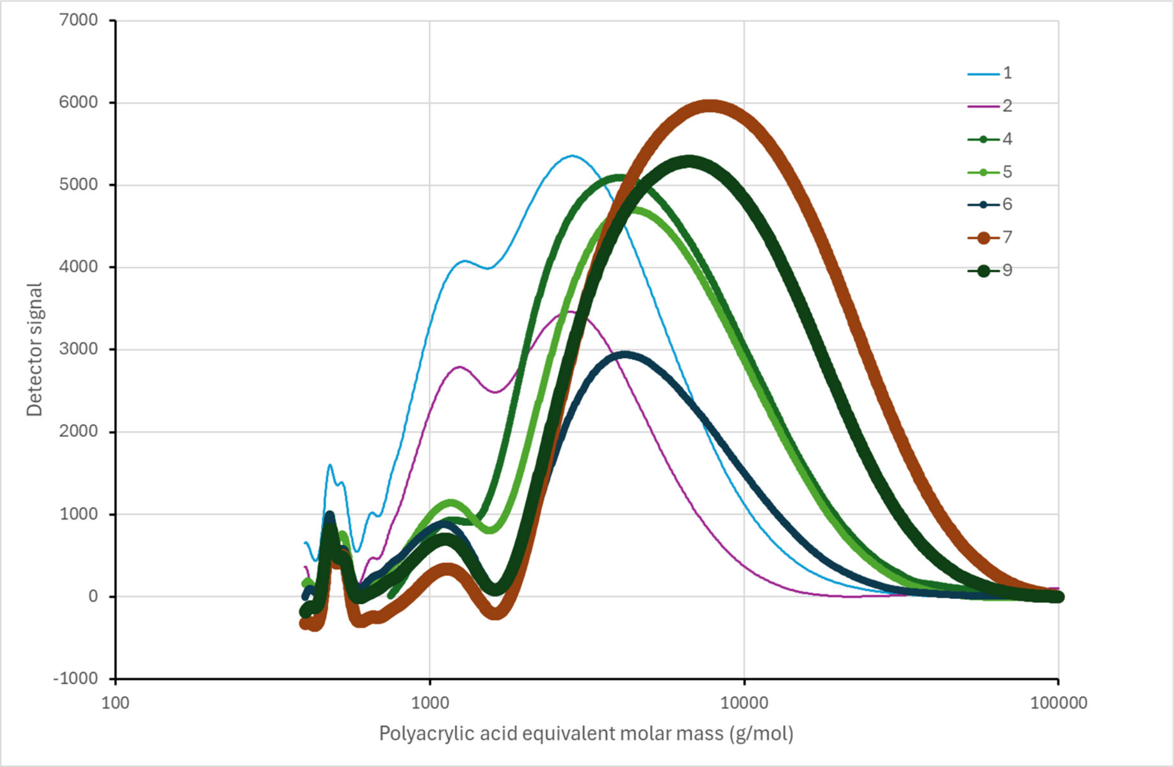

(a)

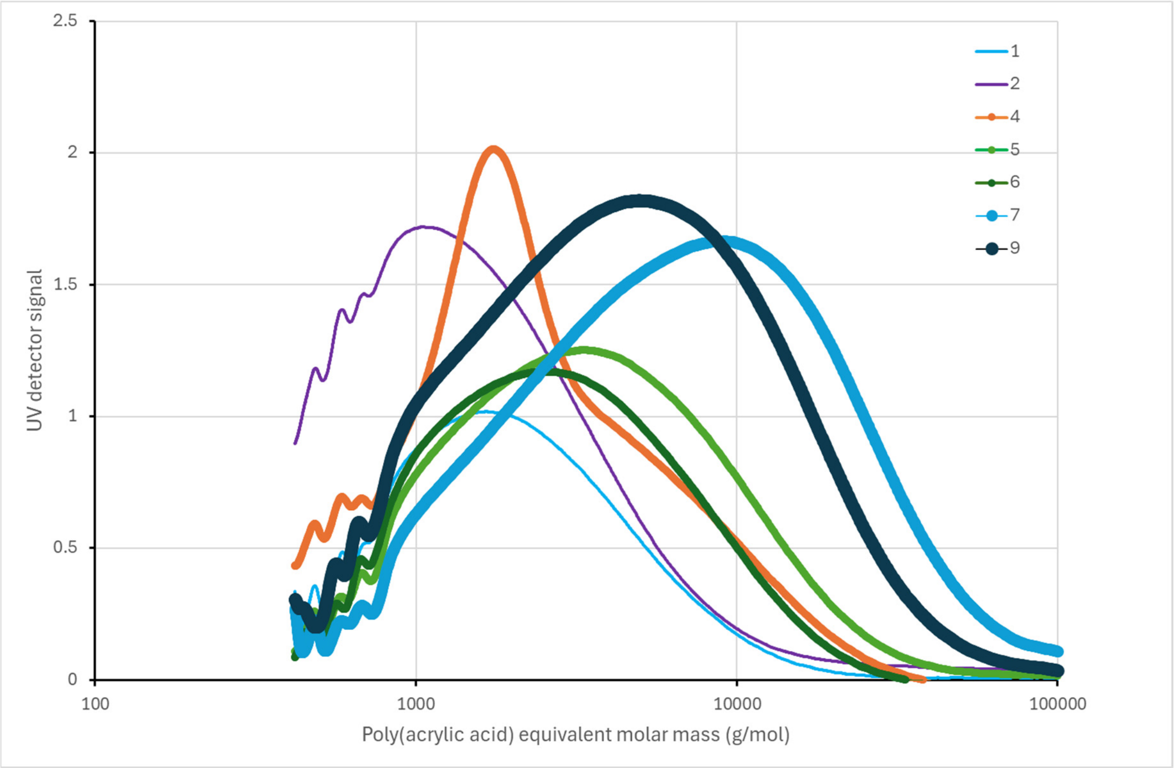

(b)

Figure S3: Size Exclusion Chromatrography eluagrams; (a) RI detector; (b) UV detector
